# Antigen-induced airway hyperresponsiveness and obstruction is related to caveolin-1 expression in airway smooth muscle in a guinea pig asthma model

**DOI:** 10.1186/s13601-015-0058-7

**Published:** 2015-03-26

**Authors:** Mayra Álvarez-Santos, Patricia Ramos-Ramírez, Fernando Gutiérrez-Aguilar, Sandra Sánchez-Hernández, Ricardo Lascurain, Raúl Olmos-Zuñiga, Rogelio Jasso-Victoria, Norma A Bobadilla, Blanca Bazan-Perkins

**Affiliations:** Instituto Nacional de Enfermedades Respiratorias Ismael Cosío Villegas, Departamento de Hiperreactividad Bronquial, Calzada de Tlalpan, 4502 Mexico; Departamento de Bioquímica, Facultad de Medicina, Universidad Nacional Autónoma de México, México, DF Mexico; Departamento de Cirugía Experimental, Instituto Nacional de Enfermedades,Respiratorias Ismael Cosío Villegas, Calzada de Tlalpan, 4502 Mexico; Molecular Physiology Unit, Instituto de Investigaciones Biomédicas, Universidad Nacional Autónoma de México, México, Mexico; Instituto Nacional de Ciencias Médicas y Nutrición Salvador Zubirán, Department of Nephrology, México, Mexico

**Keywords:** Airway hyperresponsiveness, Airway obstruction, Airway smooth muscle, Asthma, caspase 3, Caveolin-1, Cavin, Pulmonary arterial smooth muscle

## Abstract

**Background:**

Caveolin-1 is a fundamental signalling scaffold protein involved in contraction; however, the role of caveolin-1 in airway responsiveness remains unclear. We evaluated the relationship between caveolin-1 expression in airway smooth muscle (ASM) and antigen-induced airway responsiveness and obstruction in a guinea pig asthma model.

**Methods:**

Airway obstruction in sensitised guinea pigs, induced by antigenic (ovalbumin) challenges administered every 10 days, was measured. Antigen-induced responsiveness to histamine and the expression of caveolin-1 and cavin 1, 2 and 3 were evaluated at the third ovalbumin challenge. The control group received saline solution instead of ovalbumin.

**Results:**

After the first challenge, antigen exposure induced a transient airway obstruction and airway hyperresponsiveness, high levels of IL-4 and IL-5 in lung and airway globet cells proliferation at the third antigenic challenge. Caveolin-1 mRNA levels in total lung decreased in the experimental group compared with controls. Flow cytometric analysis of ASM from the experimental group showed a high number of cells expressing caveolin-1 compared with controls. This increase was confirmed by western blot. Airway obstruction and hyperresponsiveness correlated with the degree of increased caveolin-1 expression in ASM cells (*P* < 0.05; r = 0.69 and −0.52, respectively). The expression of cavins 1, 2 and 3 in ASM also increased in the experimental group compared to controls. Immunohistochemical findings reveal that differences in ASM caveolin-1 were not evident between groups. Nevertheless, a marked decrease in caveolin-1 and caspase 3 was observed in the pulmonary vascular smooth muscle of asthma model compared with controls. Histological analysis did not reveal differences in smooth muscles mass or subepithelial fibrosis levels in airways between groups. However, an enlargement of smooth muscle mass was observed in the pulmonary microvessels of experimental animals. This enlargement did not induce changes in pulmonary or systemic arterial pressures.

**Conclusions:**

Our data suggest that caveolin-1 expression in ASM has a crucial role in the development of antigen-induced airway obstruction and hyperresponsiveness in a guinea pig asthma model. In addition, the asthma model in guinea pigs appears to induce a contractile smooth muscle phenotype in the airways and a proliferative smooth muscle phenotype in pulmonary vessels.

## Background

Airway smooth muscle is a central structure in asthma pathogenesis. An important characteristic of asthma is that numerous stimuli can trigger intense and rapid bronchospasm in a phenomenon called airway hyperresponsiveness [1,2]. Currently, the precise mechanism by which the development of hyperresponsiveness is induced remains unknown. Nevertheless, airway remodelling features such as fibrosis and smooth muscle hypertrophy/hyperplasia have been recognised as playing a part [2].

Caveolin-1 is a hairpin-loop protein that forms omega-shape invaginations in the plasma membrane, which are known as caveola [3]. In asthma, a shortage of caveolin-1 has been observed in the airways of asthmatic patients [4]. Similar results have been noted in the lungs of ovalbumin-challenged mice, where a reduction of caveolin-1 mRNA expression has been observed [5,6]. In contrast, increased levels of caveolin-1 are found in the airway smooth muscle of antigen-challenged in mice [7] and the lungs of guinea pigs subjected to an asthma model [8].

In airways, caveolin-1 is involved in the downregulation of fibrosis and smooth muscle proliferation [9,10]. However, the role of caveolin-1 in airway hyper-responsiveness is unclear. The development of airway hyperresponsiveness in allergen (ovalbumin)-challenged mice without caveolin-1 has been observed [7,11], and Hsia and colleagues [12] have found the absence of caveolin-1 induced airway hyperresponsiveness in endotoxin (lipopolysaccharide)-challenged mice. Moreover, the role of caveolin-1 in airway hyperresponsiveness has become highly controversial due to the view that caveolin-1 is related to the regulation of contractile mechanisms [9,13,14], including, proteins that participate in intracellular Ca^2+^ mobilisation [15,16]. For example, M3 muscarinic, bradykinin, and H1 histamine receptors and store-operated Ca^2+^ entry-regulatory mechanisms colocalise with caveolin-1 [17]. Additionally, the recruitment of Ca^2+^ sensitisation components such as RhoA and PKCα is caveolin-1-dependent [18,19]. Furthermore, caveolin-1 is a key regulator of store-operated Ca^2+^ entry by increasing Orai1 expression in airway smooth muscle [20].

Since 2005 some proteins named cavins has been associated with caveola biogenesis and organization [21]. In particular, cavin 1 (RNA pol I transcription factor), cavin 2 (serum deprivation protein response) and cavin 3 (SDR- related gene product that binds to C kinase) are widely expressed in tissues, included smooth muscles [22]. Recently, it has been observed a decrease in expression of cavins in airways of caveolin-1 knock-out mice, although its role in airway contraction its unknown [7].

Experimental asthma models are fundamental in asthma research. Particularly, guinea pigs asthma model are susceptible to develop early and late allergic responses after allergen challenge and also can be used as a model for chronic allergic asthma [23,24]. Asthma model in guinea pig is useful since the lung pharmacology and the response to inflammatory mediators is similar to humans in comparison to rats and mouse [25,26].

In the current study, we determined the relationship between caveolin-1 expression and the pathophysiological characteristics of asthma and found that caveolin-1 expression increases in airway smooth muscle and that this increase is related to antigen-induced obstruction and hyperresponsiveness. In contrast, pulmonary vascular smooth muscle showed low expression of caveolin-1, which was accompanied by smooth muscle cell proliferation.

## Methods

We used outbred male guinea pigs weighing 0.35-0.4 kg from Harlan Mexico (strain HsdPoc:DH). The animals were maintained in our institutional laboratory animal facilities with filtered air conditioned at 21 ± 1°C and 50-70% humidity, 12/12-h light/dark cycles, sterilised pellets (2040 Harlan Teklad Guinea Pig Diet) and water available *ad libitum*. All animals were handled according to protocols approved by the Scientific and Bioethics Committee of the Instituto Nacional de Enfermedades Respiratorias.

### Study design

**Figure 1:**
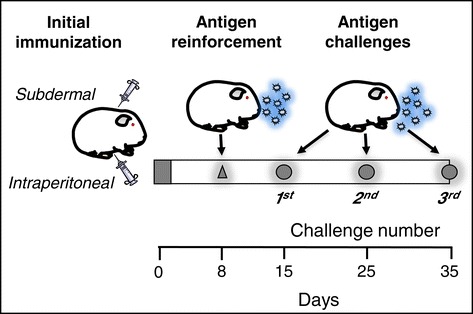
**Experimental design.** After initial immunisation and reinforcement with antigen (ovalbumin), guinea pigs received three antigen challenges. At third challenge the evaluation of broncho-obstructive index, dose–response curves to histamine as well as immunological, histopathological and vascular function analysis were performed.

To determinate the role of caveolin-1 in airway smooth muscle pathophysiology during asthma, ovalbumin sensitised guinea pigs were exposed to three antigenic challenges, each administrated every 10 days (Figure 1). During each challenge, the broncho-obstructive index was measured. At the third antigenic challenge, the development of antigen-induced airway hyperresponsiveness was evaluated by performing dose–response curves to histamine before and after an antigenic challenge. Animals were then sacrificed to obtain lung and tracheal samples. In lung samples, caveolin-1 mRNA was measured by RT-PCR. Additionally, changes in the amount of collagen in the airway lamina propria and the extent of airway and pulmonary microvessel smooth muscle layers were analysed via light microscopy. Caveolin-1 expression was examined using immunohistochemistry. Caveolin-1 expression in smooth muscle cells from tracheae was measured by flow cytometry. Tracheal smooth muscle strips were used to evaluate the expression of caveolin-1 and cavins 1, 2 and 3 by western blot. Systemic and pulmonary arterial pressures were measured at the third challenge. Control animals received sham manoeuvres performed with saline solution.

### Asthma model

Guinea pigs were sensitised and challenges were performed according to previously described methods [23,27]. The antigen sensitisation of guinea pigs was performed by intraperitoneal (0.5 mg/ml) and subdermal (0.5 mg/ml) injections with a combination of 60 μg/ml ovalbumin plus 1 mg/ml aluminium hydroxide dispersed in saline solution (Figure 1). The doses used in sensitisation and challenges in this asthma model were adjusted to reduce anaphylactic shock during challenges. Antigen sensitisation was reinforced eight days later with ovalbumin aerosol (3 mg/ml saline) delivered over five minutes. Aerosols were produced by a US-1 Bennett nebuliser (flow, 2 ml/min; Multistage liquid impinger, Burkard Manufacturing Co., Rickmansworth, Hertfordshire, UK) releasing mixed particles with sizes of <4 μm (44%), 4–10 μm (38%), and >10 μm (18%). From day 15 onward, guinea pigs were challenged over one minute with an ovalbumin aerosol every 10 days (1 mg/ml during the first challenge and 0.5 mg/ml in subsequent challenges) (Figure 1).

Acute airway obstructive responses after ovalbumin inhalation challenges were recorder using a barometric plethysmograph. A whole-body single-chamber plethysmograph for freely moving animals was used (Buxco Electronics Inc., Troy, NY, USA) to evaluate pulmonary function. The signal from the chamber was processed with computer-installed software (Buxco Bio System XA v1.1) to calculate several respiratory parameters, including the broncho-obstructive index, Penh. We calculated this index using the following equation [28]:

### Penh = ((Te-Rt)/Rt) (PEP/PIP)

where Te = expiratory time (s), Rt = relaxation time (s), PEP = peak expiratory pressure (cmH_2_O), and PIP = peak inspiratory pressure (cmH_2_O). The software was adjusted to include only breaths with a tidal volume of 1 millilitre or more, with minimal inspiratory time of 0.15 seconds, maximal inspiratory time of 3 seconds, and maximal difference between inspiratory and expiratory volumes of 10%. This guinea pig model of allergic asthma does not develop a noticeable late airway response. We corroborated that this sensitisation procedure induces the increment of Th2 (CD4 + IL13+) lymphocytes in bronchoalveolar lavage.

### Antigen-induced airway responsiveness

In guinea pigs, airway hyperresponsiveness was measured after antigen challenge in sensitised (*n* = 18; asthma model) and non-sensitised (*n* = 13; control group) animals [27]. Airway responsiveness was evaluated on day 35 (third ovalbumin challenge) by exposing each animal to increasing non-cumulative doses of histamine aerosols (0.001 to 0.32 mg/ml; Sigma Chemical Co., St. Louis, MO, US) after an initial broncho-obstructive index acquisition before and after ovalbumin administration. Each histamine dose was delivered over 1 minute, and the average of the broncho-obstructive index over the following 10 min was obtained. The interval between doses was 10 min. The dose–response curve finished when the broncho-obstructive index reached three times its baseline level. Once the index returned to the initial baseline value (<50% increment), the ovalbumin challenge was administered. A second curve was measured three hours later. Control group received saline instead ovalbumin administration.

### Dissection of airway smooth muscle strips for flow cytometry and Western blot studies

Twenty-four hours after concluding histamine curves, some sensitised (*n = 10)* and non-sensitised (*n = 9)* guinea pigs were overdosed with an intraperitoneal injection of pentobarbital sodium (65 mg/kg), and their tracheae were dissected to obtain airway smooth muscle strips.

### Isolation of airway smooth muscle cells

The strips of 7 sensitised and 6 non-sensitised guinea pigs were incubated at 37°C for 10 min in 5 ml of Hanks’ solution (Gibco, Gaithersburg, MD, US) with 2 mg cysteine and 0.05 U/ml papain (Sigma Chemical Co., St. Louis, MO, US). The strips were then washed in Leibovitz’s solution (Gibco, Gaithersburg, MD, US) and placed in a physiological saline solution (PSS, mM) containing 118 NaCl, 25 NaHCO_3_, 4.6 KCl, 1.2 MgSO_4_, 1.2 KH_2_PO_4_ and 11 glucose (Sigma Chemical Co., St. Louis, MO, US). Smooth muscle strips were cut (0.5 x 5 mm), and fragments weighing 200 mg total were placed in 2.5 ml PSS with collagenase type I (1 mg/ml; Sigma Chemical Co., US) and dispase II (4 mg/ml; Sigma Chemical Co., St. Louis, MO, US) at 37°C. Ten minutes later, the fragments were transferred to similar PSS containing fresh enzymes. Tissue was dispersed mechanically until isolated cells were observed. Leibovitz’s solution was added to stop the enzymatic activity.

### Flow cytometry

For the detection of caveolin-1 production in the isolated airway smooth muscle cells, a three-color immunofluorescence approach was used following a previously described method [29]. Isolated myocytes were incubated with 10 μg/ml brefeldin-A (Sigma Chemical Co.; St. Louis, MO, US) for four hours to inhibit new cytokine release. After staining, cells were washed, fixed with 4% p-formaldehyde for 10 min at 4°C, washed, and permeabilised with 0.1% saponin in PBS with 10% BSA and 1% NaN_3_. Afterwards, cells were gently shaken in the dark for 15 min at room temperature and 1 μl/1x10^6^ cells were labelled with caveolin-1 antibody (BD Biosciences Pharmingen, San Diego, CA, US). Then, cells were incubated during 30 min with secondary antibody FITC mouse (BD Biosciences Pharmingen, San Diego, CA, US). Finally, cells were analysed for the expression of markers, on a FACScan flow cytometer (Becton Dickinson, San Diego, CA, US) using software, and 10,000 events were counted. To analyse the staining of intracellular caveolin-1, the blasts were initially gated by their physical properties (forward and side scatter). A second gate was then drawn based on the fluorescence characteristics of the gated cells, assessing fluorescence intensity by histograms. Intensity of fluorescence staining is expressed as the mean fluorescence intensity. Control stains were performed using fluorochrome-conjugated isotype-matched antibodies. Background staining was <1% and subtracted from experimental values.

### Western blot analysis

Smooth muscle strips from guinea pig tracheae (*n* = 3, each group) were placed in lysis buffer (1% Triton X-100, 50 mM Tris, pH 7.4, 150 mM NaCl, 0.1 mM EDTA and EGTA, 1.0 mM phenylmethylsulfonyl fluoride, 10 μg/ml aprotinin and leupeptin, 1.0 mM Na_3_VO_4_, and 50 mM NaF; Sigma Chemical Co.; St. Louis, MO, US) and homogenised (Polytron PT3100, Kinematica, Switzerland). Tissue protein (40 μg) from each sample was loaded in different lanes of a 12% SDS-polyacrylamide gel. In an additional lane, a control protein (GAPDH; Sigma Chemical Co.; US) was also added. After electrophoretic separation under reducing conditions, proteins were transferred to a nitrocellulose membrane and quenched with Tris-buffered saline (TBS) containing 5% non-fat milk and 0.1% Tween-20. Membranes were subjected to overnight incubation (12 h, 4°C) with rabbit polyclonal antibodies raised against caveolin-1 (BD Biosciences Pharmingen, San Diego CA, US), cavin-1 (Anti/PTRF/Cavin-1, Millipore, Billerica MA, USA), cavin-2 (SDR, Thermo Scientific, Rockford IL, USA) and cavin-3 (PRKCDBP, Thermo Scientific, Rockford IL, USA), and then washed three times with TBS-Tween-20 (0.1%). Caveolin and cavins were detected by adding horseradish peroxidase-labelled anti-mouse antibodies. Immunoblots were developed using an enhanced chemiluminescent reactant (LumiGLO, Cell Signalling; US) and an optimal exposition of the nitrocellulose sheets to X-ray films (Biomax ML Film, Kodak, Rochester, NY US). Caveolin-1 and cavin immunoblots were analysed by densitometry using Kodak digital science ID software version 2.03 (Eastman Kodak, Rochester, NY, US).

### RNA isolation

Total RNA from the right lung of some sensitised and non-sensitised (*n* = 3, each group) guinea pigs was isolated following the guanidine isothiocyanate-caesium chloride method [30]. Total RNA was examined by 1% agarose gel electrophoresis, and the RNA concentration was determined by UV light absorbance at 260 nm to evaluate the integrity of RNA (Beckman DU640, Fullerton, CA, US).

### RT-PCR

The relative level of caveolin-1 mRNA expression was assessed in left lung homogenates by semiquantitative RT-PCR, as previously described [31]. Briefly, all primer sequences were custom ordered from GIBCO BRL (Gaithersburg, MD, US). Sense caveolin-1 primers were amplified to obtain a fragment of 230 bp, bases 1 to 230 (sense 5′-ATG TCT GGG GGT AAA TAC GT-3′ and antisense: 5′-CCT TCT GGT TCC GCA ATC AC-3). A fragment of GAPDH was also amplified to evaluate or reduce nonspecific effects of experimental treatment and to semi-quantify caveolin-1 expression. RNA samples were treated with DNAse to evaluate genomic DNA contamination relative to samples passed through a PCR procedure without adding reverse transcriptase. RT-PCR was carried out using 2.5 μg of total RNA from lung homogenate. Before the RT-PCR reaction, total RNA was heated at 65°C for 10 min. RT-PCR was performed at 37°C for 60 min in a total volume of 20 μl using 200 U of the Moloney murine leukaemia virus reverse transcriptase (GIBCO BRL, Gaithersburg, MD, US), 100 pM of random hexamers (GIBCO, BRL Gaithersburg, MD, US), 0.5 mM of each dNTP (Sigma, St. Louis, MO, US), and 1× RT buffer (75 mM KCl, 50 mM Tris · HCl, 3 mM MgCl_2_, 10 mM DTT, pH 8.3). Samples were heated at 95°C for 5 min to inactivate the reverse transcriptase and diluted to 40 μl with PCR-grade water. One-tenth of RT-PCR individual samples from each group was used for caveolin-1 or GAPDH amplification in 20-μl final volume reactions containing 1× PCR buffer (10 mM Tris · HCl, 1.5 mM MgCl_2_, 50 mM KCl, pH 8.3), 0.1 mM of each dNTP, 0.2 μCi of [α32P]-dCTP (3,000 Ci/mmol, 9.25 MBq, 250 μCi), 10 μM of each primer, and one unit of Taq DNA polymerase (GIBCO, BRL Gaithersburg, MD, US). Samples were overlaid with 30 μl of mineral oil and PCR cycles were performed in a DNA thermal cycler (M.J. Research, Watertown, MA, US), with the following profile: denaturation for 1 min at 94°C; annealing for 1 min at 55°C and a 1-min extension step at 72°C. The last cycle was followed by a final extension step of 5 min at 72°C. Control gene was co-amplified simultaneously in each reaction. Amplification kinetics were performed following our standard procedure [31]. To analyse PCR products, one-half of each reaction was electrophoresed in a 5% acrylamide gel. Bands were stained with ethidium bromide, visualised under UV light, cut out, suspended in 1 ml of scintillation cocktail (Ecolume, ICN, Aurora, OH, US), and counted by liquid scintillation (Beckman LS6500, Fullerton, CA, US). The amount of radioactivity recovered from the excised bands was plotted in a log scale against the number of cycles. To semi-quantify caveolin-1 and the control gene, all reactions were performed at least in quadruplicate.

### Conventional histology and automated morphometry analysis

Left caudal lung lobes of some guinea pigs (*n* = 6, each group) were dissected and fixed by manual perfusion of 10% neutral buffered formaldehyde solution via intra-arterial route until the lung lobe was exsanguinated. Lung fragments obtained by sagittal cutting were embedded in paraffin, and 4 μm-thick lung sections were stained with Masson trichrome stain. The surface areas (μm^2^) of airway smooth muscle and lamina propria, as well as the vascular smooth muscle of adjacent vessels, were determined through the use of automated morphometry (Qwin, Leica Microsystems Imaging Solutions, Cambridge, UK). Data were adjusted by length of the corresponding basement membrane, and their average was considered the final result. All measurements were conducted in six bronchi, six bronchioles and six arterioles (~100 μm diameter) chosen randomly from each animal. Total epithelial cells in six bronchi of each guinea pig were counted and the percentage of globet cell was obtained. The bronchus and bronchiole were identified by the presence or absence of cartilage in the airway wall, respectively.

### ELISA

Anti-human interferon-γ (IFN-γ; R&D System, Minneapolis, USA), interleukin-4 (IL-4; R&D System, Minneapolis, USA) and IL-5 (clone TRFK5; BD Pharmingen, USA), antibodies were used in lung homogenates of sensitized and non sensitised guinea pigs (*n* = 6, each group) to measure cytokines by ELISA as previously described [27].

### Immunohistochemistry and immunofluorescence

The same paraffin-embedded lung tissue blocks used for the morphometric study were used for immunohistochemistry (*n* = 6, each group) and immunofluorescence (*n* = 2, each group). Sections (3 μm) were deparaffinised (55°C, 30 min) and rehydrated through submersion in graded alcohols (xylene, 1:1 xylene-alcohol, alcohol, and 70% alcohol for 10 min each, followed by rinsing in distilled water). Antigen retrieval was performed with 10 mM citrate buffer, pH 6, for 5 min in a microwave oven. Samples were treated with hydrogen peroxide (3%) to quench endogenous peroxidase, and nonspecific sites were blocked later with horse serum (2%). Sections were incubated at 4°C overnight with an antibody to caveolin-1 (BD Biosciences Pharmingen, San Diego CA, US). To detect the specific binding of this primary antibody, an R.T.U. Vectastain Universal Quick Kit was used (Vector Laboratories, Inc., Burlingame, CA, USA) in which tissues were incubated sequentially with blocking serum, a pan-specific secondary antibody, and streptavidin/peroxidase complex. Finally, 3-amino-9-ethyl-carbazole (BioGenex, San Ramon, CA, USA) was used as a chromogen. Sections were counterstained with Mayer’s haematoxylin. Slides were rinsed twice with 0.1% Tween-20 phosphate-buffered saline during the whole process. To control for the non-specific binding of the secondary antibody, sections from the same lung were processed without the primary antibody. No positive staining was observed in non-specific binding controls. The rabbit IgG (Southern Biotech, Birmingham, AL, USA) isotype control was negative.

For the detection of caspase 3 by immunofluorescence, paraffin-embedded tissue was cut 4–6 m thick and tissue sections were placed in slides and incubated for 30 min at 55°C. Tissue slides were deparaffinized in xylenes twice for 10 min each. Hydrate sections gradually through graded alcohols using 2 changes for 10 min each of the following solutions: 100% ethanol, 95% ethanol, and deionized water. For antigen unmasking, slides were covered with 10 mM sodium citrate buffer, pH 6.0, and heat at 95°C for 5 min. Slides were then cooled in TBS-T buffer for 20 min at room temperature. To suppress non-specific binding of antibodies, tissue slides were incubated with 0.2% BSA in PBS for 20 min at 4°C. After that, immunofluorescence staining was carried out by overlaying each slide with 20 μl of rabbit polyclonal anti-caspase 3 antibody (Abcam, San Francisco, CA, USA) for 2 h at 4°C. After washing in TBS-T buffer for 5 min, a secondary incubation was performed with fluorescein isothiocyanate (FITC)-labeled goat anti-rabbit IgG (Jackson ImmunoResearch Laboratories Inc. Amish Country, PA, USA) for 30 min at 4°C. After twice washing, slides were counterstained with fluoroshield mounting medium with DAPI (Sigma-Aldrich Co, St. Louis, MO, USA) for nuclei staining (blue channel). All incubations were carried out in a humidified dark chamber. In other experiments, tissue slides were incubated only with FITC-labeled secondary antibody, which was used as background staining control. Finally, tissue slides were examined by fluorescence microscope with appropriate filters (Leica DM-LS 2000, Mannheim, Germany), and analyzed by ImageJ64 software (http://rsb.info.nih.gov/ij/).

### Hemodynamic

After 12 h of fasting, some guinea pigs (*n* = 8 for sensitised, and *n* = 4 for non-sensitised) were anaesthetised with isofluorane (1.5%, Sofloran, México, DF) delivered by a precision vaporiser (Isotec 3, Ohmeda, Steeton, West Yorkshire, UK) carried in oxygen. Their heart rates were monitored with an automatic non-invasive device Datascope Passport Model EL (Datascope Corp, Mahwah, NJ, USA), and after deep anaesthesia, animals were ventilated through a trachea cannula that was connected to both, a ventilator (Harvard, Rodent Model Ventilator 683) and the vaporiser. Then, the right carotid artery was dissected, and a catheter was introduced to measure the systemic arterial pressure and diastolic arterial pressure. A similar procedure was performed to introduce a catheter into the pulmonary artery through the right ventricle to determine the pulmonary arterial pressure. Datascope Passport monitored all pressures.

## Materials

Ovalbumin (chicken egg albumin) grade II and all stains for microscopy were purchased from Sigma Chemical Co. (US). Aluminium hydroxide was purchased from J.T. Baker, USA. Pentobarbital sodium was acquired from Pfizer, Mexico.

### Statistical analysis

Airway responsiveness to histamine was evaluated by means of the provocative dose 200% (PD_200_), i.e., the interpolated histamine dose that caused a three-fold increase of basal broncho-obstructive index. Change in histamine responsiveness induced by antigen challenge was evaluated by the PD_200_ ratio, i.e., PD_200_ value observed after OVA challenge divided by PD_200_ value before challenge. In multiple comparisons, one-way or repeated-measure ANOVA followed by Dunnett’s tests was used. Comparison between control and asthma model groups was evaluated by means of Student’s unpaired *t*-test. Associations between caveolin-1 and airway responsiveness and obstruction were assessed through Spearman’s correlation coefficient. Statistical significance was set at two-tailed *P* < 0.05. Data in the text and figures are expressed as the mean ± SEM.

## Results and discussion

### Antigen-induced airway obstruction and responsiveness

**Figure 2:**
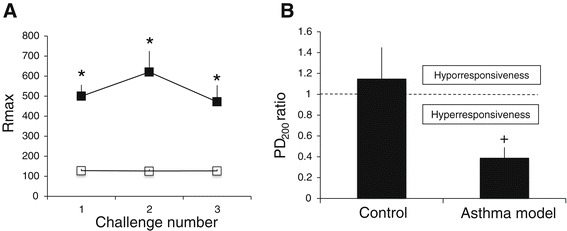
**Antigen-induced airway obstruction and responsiveness in sensitised guinea pigs. A**) Average of maximum broncho-obstructive index (Rmax) induced by ovalbumin (closed squares) and saline (open squares) challenges in sensitised guinea pigs. **B**) PD_200_ ratio corresponds to PD_200_ value observed after antigen challenge divided by PD_200_ value before challenge. Bars represent mean ± SEM (*n* = 7 per group). *P < 0.05 compared with control (repeated measures ANOVA with Dunnett’s multiple comparisons test). ^+^ *P* < 0.05 compared with control (unpaired Student’s *t*-test).

The guinea pig has served as a helpful model in the study of asthma, with some advantages over other models such as the rat and mouse, because it shares various pharmacological characteristics of human asthma, besides the strong airway obstructive response that guinea pigs exhibit after agonist stimulation [32]. Responses to antigen challenge in guinea pig asthma models are characterised by a rapid and transient airway obstruction in sensitised animals [23,27]. In the current study, the values of basal airway obstruction index were similar between control and experimental groups (data not shown). Saline challenge did not modify the basal obstruction index in the control group, but ovalbumin challenge induced a transient increase in the index that reached statistical significance in comparison with the control group (*P* < 0.05; *n* = 18 asthma model, and *n* = 13 control group; Figure 2A).

An important pathophysiological feature of asthma is the development of airway hyperresponsiveness. In this study, the basal histamine PD_200_ value was similar between the control and experimental groups (data not shown). In the control group, the histamine PD_200_ value after saline challenge was similar to the basal PD_200_ value. In the experimental group, the PD_200_ value after ovalbumin challenge was lower than the basal PD_200_ value. In the control group, the PD_200_ ratio was significantly lower than that obtained in the control group (*P* < 0.05; *n* = 18 asthma model, and *n* = 13 control group; Figure 2B), implying that all guinea pigs sensitised with the antigen showed hyperresponsiveness to histamine.

**Figure 3:**
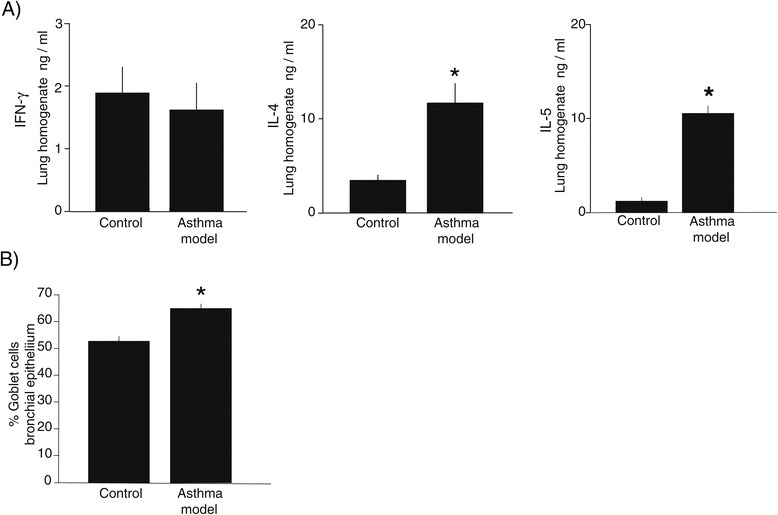
**Cytokine expression and globet cells percentage in sensitized guinea pigs. A**) Levels of interferon-γ (IFN-γ), interleukin (IL) -4 and IL-5 in lung homogenate; **B**) Globet cell percentage in bronchi. The bars represent the means ± SE of *n* = 6 guinea pigs in each group. *P < 0.05 compared with control (unpaired Student’s *t*-test).

Inflammatory markers in asthma as the Th2 cytokines IL-4 and IL-5 [33] significantly increases in lung homogenates of asthma model guinea pigs in comparison with controls (*P* < 0.05; *n* = 6 each group; Figure 3A). In contrast IFN**-**γ, a Th1 cytokine, has similar levels in both groups (Figure 3A). In addition, a significantly increment of globet cells was observed in bronchi epithelium of asthma model group (P < 0.05; *n* = 6 each group; Figure 3B) suggesting that this model of acute asthma induces structural changes in airway epithelium.

### Caveolin-1 expression in asthma model

In comparison with other cells, smooth muscle expresses high levels of caveolin-1 in the plasma membrane [34]. In airway smooth muscle, the signalling platform associated with caveolin- 1 takes important roles in the recruitment of various signalling proteins involved in contraction. For example, caveolin-1 is involved in Ca^2+^ homeostasis via the presence of voltage gated l-type Ca^2+^ channels, the plasma membrane Ca^2+^ ATPase, calsequestrin and calreticulin in caveolin-enriched membranes [15]. Other components that play a role in airway smooth muscle contraction, such as M3 muscarinic, bradykinin, H1 histamine, phospholipase Cβ1, Gαq and store-operated Ca^2+^ entry-regulatory mechanisms, co-localise with caveolin-1 [17,35]. Moreover, TNF-α, a fundamental cytokine in asthma pathogenesis [36], induces RhoA activation, enhances force responses to acetylcholine, and increases Ca^2+^ responses to acetylcholine, histamine and bradykinin through caveolin-1 upregulation [18,37,38]. Certainly, caveolin-1 is associated with key molecules that participate in airway smooth muscle contraction.

**Figure 4:**
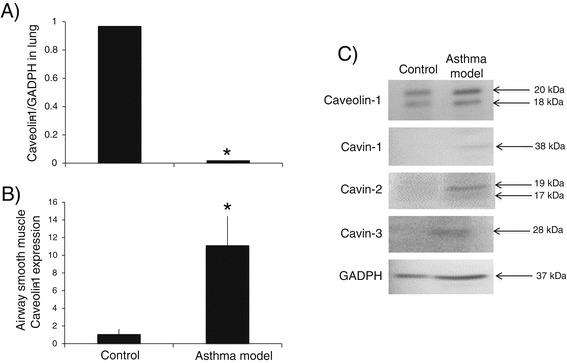
**Caveolin-1 expression in airway smooth muscle. A**) Caveolin-1 mRNA levels in lung (*n* = 3 in all groups). **B**) Caveolin-1 expression in tracheal isolated airway myocytes, measured by flow cytometry (*n* = 6, 7 for control and asthma model groups). **C**) Representative autoradiographs of caveolin-1 and cavins-1, 2 and 3 by western blot in tracheal airway smooth muscle (*n* = 3 in all groups). Bars represent mean ± SEM. *P < 0.01 compared with control (unpaired Student’s *t*-test).

In agreement with other studies [4-6], we found that caveolin-1 is downregulated in the lung homogenates of experimental animals, as shown in Figure 4A. The level of caveolin-1 mRNA in the lung homogenate of controls was significantly higher in comparison to the experimental group (*P* < 0.01; *n* = 3 in all groups); nevertheless, flow cytometric studies in isolated myocytes demonstrated that the number of cells that express caveolin-1 increased significantly in the experimental group compared to controls (*P* < 0.01; *n* = 6 and 7 for control and asthma model groups, respectively; Figure 4B). Similar results in the airway smooth muscle bundles of experimental group animals obtained by immunohistochemistry analysis have been described previously [8]. In our study, we observed that functional changes in the asthma model correlated with the number of smooth muscle cells that expressed caveolin-1. The PD_200_ ratio was inversely correlated with airway smooth muscle caveolin-1 (r = −0.517, *P* < 0.05; *n* = 13), implying that a greater number of cells positive for caveolin-1 corresponded to greater antigen-induced airway responsiveness. In addition, the Rmax correlated with the number of cells positive for caveolin-1 (r = 0.691, *P* < 0.01; *n* = 13), indicating that antigen-induced airway obstruction is directly associated with caveolin-1 in airway smooth muscle cells.

Western blot analysis detected two specific bands for caveolin‑1 at approximately 18 to 20 kDa, and the expression of both bands increased in the experimental group (Figure 4C; *n* = 3 all groups). In addition, cavins 1, 2 and 3, a group of proteins that, along with caveolin-1, regulates caveolae organisation and function [3], were found in controls but were more highly expressed in the experimental group (Figure 4C; *n* = 3 in all groups). Previously, cavins 1, 2 and 3 have been found to increase in the airway smooth muscle of ovalbumin-sensitised mice [7]. Although the role of cavins in asthma is unknown, TNF-α induced the upregulation of cavins in airway smooth muscle, suggesting that inflammation may regulate cavin expression [39].

**Figure 5:**
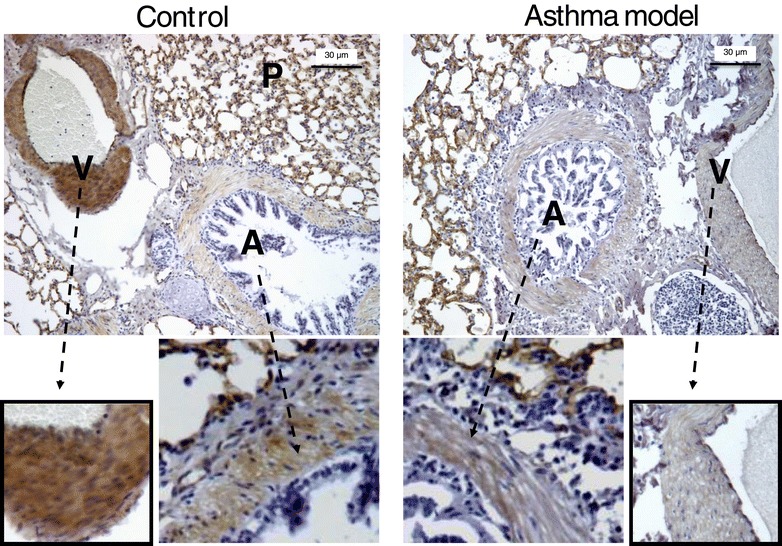
**Determination of caveolin-1 expression in control and asthma model guinea pigs by immunohistochemistry.** Upper images (4x) are representative sections of control and asthma model guinea pigs exhibiting caveolin-1 immunostaining in an airway (A) and lung vessel (V). An amplification of airway and lung vascular smooth muscles is shown in bottom images with arrows. Figures are representative of 6 guinea pigs in each group.

Immunohistochemical images did not reveal noticeable changes in caveolin-1 staining between controls and asthma model groups in airway smooth muscle or parenchyma (Figure 5; *n* = 6 all groups); nevertheless a strong reduction of caveolin-1 expression in vascular smooth muscle of the experimental group was observed in comparison with controls (Figure 5). In view that only pulmonary vascular smooth muscle showed a strong reduction in caveolin-1 expression in asthma model, and the other structures did not exhibit changes, it is possible that the intense decrease in caveolin-1 mRNA levels in total lung homogenates (Figure 4A) of the asthma model was likely produced by the downregulation of caveolin-1 in this smooth muscle.

### Airway and lung vascular smooth muscle structure

Airway remodelling has been proposed as an important factor associated with caveolin in the development of airway hyperresponsiveness. For example, in a previous study [23] of a model of chronic asthma in guinea pigs (nine antigen challenges), we found an association between subepithelial fibrosis and airway hyperresponsiveness. Caveolin-1 contributes to remodelling by producing suppressive effects in airway smooth muscle proliferation [9], likely through the inhibition of constitutive p42/p44 MAPK activity [40] and by inducing type I collagen in lung tissue [5]. Airway hyperresponsiveness is a consequence of airway remodelling induced by the lack of caveolin-1 in knock-out mice [7,11].

**Figure 6:**
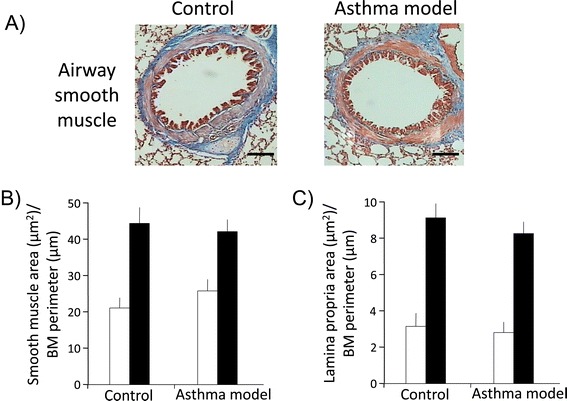
**Representative histological features of airway in a guinea pig allergic asthma model. A**) Low-power micrograph (10x) from the lungs of control and experimental guinea pigs showing bronchi. **B**) Area of smooth muscle layer and **C**) lamina propria of bronchioles (white bars) and bronchi (black bars), adjusted by the basement membrane (BM) perimeter, as measured by automated morphometry. Bars and vertical lines are mean ± SE of *n* = 6 per group. *P < 0.01 (unpaired Student’s *t*-test).

**Figure 7:**
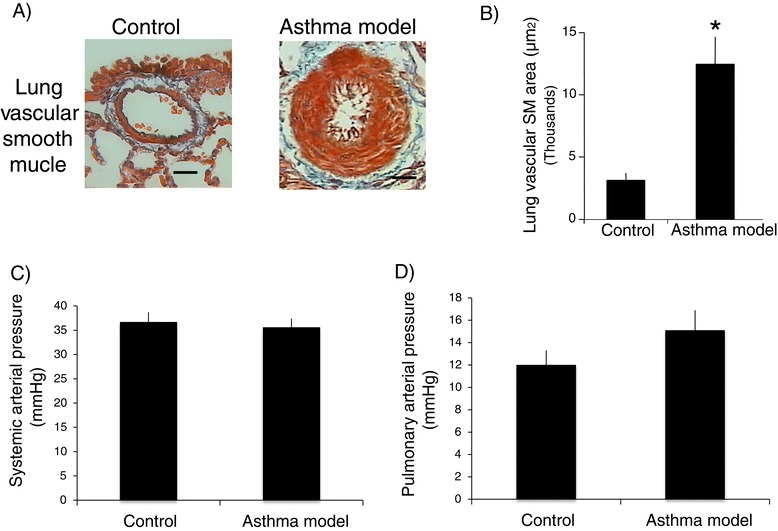
**Representative histological features of lung vascular smooth muscles in a guinea pig allergic asthma model. A**) Low-power micrograph (10x) from the lungs of control and asthma model guinea pigs showing a bronchus and an arteriole. Note the extensive smooth muscle (SM) area in the asthma model arteriole. **B**) Area of arteriole SM layer, as measured by automated morphometry (*n* = 6 per group). **C**) Systemic arterial pressure and **D**) pulmonary arterial pressures (*n* = 4 and 8 guinea pigs in control and asthma model group, respectively). Bars and vertical lines are mean ± SE. *P < 0.01 (unpaired Student’s *t*-test).

**Figure 8:**
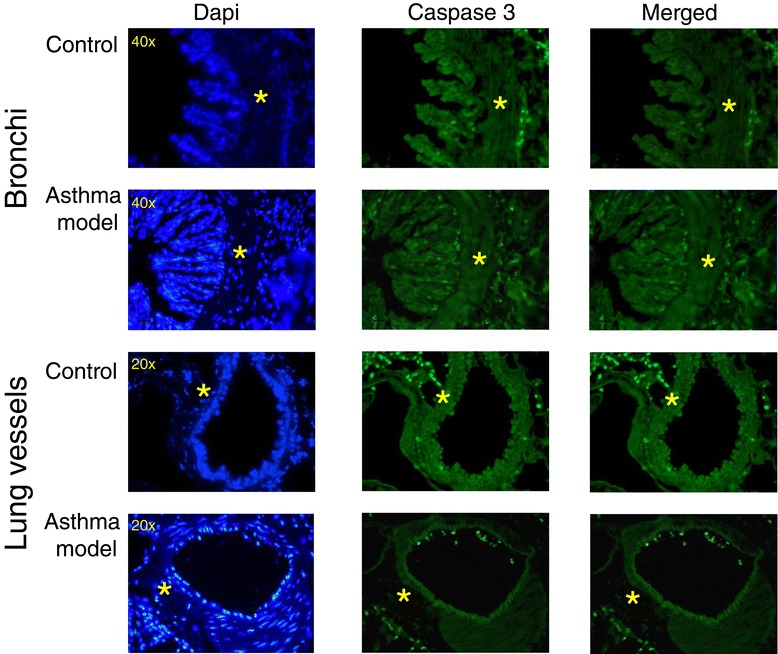
**Immunofluorescence microscopy showed a significant reduced fluorescent staining for caspase 3 in smooth muscle from blood vessels**. Lung tissue sections from guinea pig were incubated with rabbit polyclonal anti-caspase 3 antibody, followed by FITC-labeled goat anti-rabbit IgG. Nuclei were counterstained with DAPI and they are shown in blue. Caspase 3 positive (apoptotic) cells are uniformly distributed in bronchi either in samples from model asthma or control (upper panels observed by 40x magnification) as well as in blood vessel from control (medium panel, 20x magnification). In contrast, a significant decrease of apoptotic cells in blood vessel smooth muscle cells is observed in asthma model. Representative images of at least two independent experiments. * corresponds to smooth muscle bands in bronchi and lung vessels.

To evaluate airway remodelling in guinea pigs, Masson trichrome staining was used to distinguish airway and lung vascular smooth muscle layers based on their strong red cytoplasmic staining (Figures 6A and 7A). Automated morphometric analysis of smooth muscle mass and subepithelial fibrosis of bronchioles and bronchi did not show significant differences between control and asthma models (Figure 6B and C; *n* = 6 in control and *n* = 7 in asthma model groups). In addition, we did not observe an association between the degree of hyperresponsiveness and the level of subepithelial fibrosis (r = 0.25; *n* = 13) or airway smooth muscle mass (r = 0.18; *n* = 13). Nevertheless, widening of the airway adjacent vessels was observed (*P* < 0.05; *n* = 6 per group; Figure 7A and B). The total numbers of smooth muscle nuclei in bronchioles in the experimental and control groups were similar (29 ± 2.3 and 28 ± 2.5 nuclei, respectively; *n* = 6 per group, data not illustrated); however, an increase in the number of total smooth muscle cell nuclei from airway adjacent vessels was observed in experimental guinea pigs compared with controls (51 ± 10.4 and 12 ± 1.2 nuclei, respectively; *P* < 0.01; *n* = 6 per group, data not shown). In addition, caspase 3 expression determinate by immunofluorescence in bronchi smooth muscle did not show noticeable change in asthma model and control groups; nevertheless, an evident diminution of caspase 3 expression was observed in lung smooth muscle from vessels in asthma model group (*n* = 2; Figure 8). It suggests that apoptosis is inhibited in vascular smooth muscle in asthma model, a tissue that also shows a decrement of caveolin-1 expression (Figure 5) and an enlargement of tissue area (Figure 7B). According to the above, these findings are in agreement with the antiproliferative and proapoptotic effects induced by caveolin-1 observed in almost cell types [41]. Then, in conclusion these results suggest that pulmonary vascular smooth muscle, but not airway smooth muscle, is capable to show remodelling changes in acute asthma model in guinea pigs and that caveolin-1 is associated with this phenomenom.

### Arterial pressure in asthma model

A feature of the contractile, but not proliferative, airway smooth muscle phenotype is the abundance of caveolae, suggesting that caveolin-1 may have an inhibitory role in airway smooth muscle proliferation [9]. In contrast, the intense diminution of caveolin-1 observed in smooth muscle from airway adjacent vessels in experimental guinea pigs appears to be related to the increase in proliferation. In this sense, it is known that caveolin-1 has a fundamental role in regulating the proliferation of vascular smooth muscle [42]. In mice, the absence of caveolin-1 has been shown to modify arterial filling and increase pulmonary vascular resistance [43]. To determinate the putative physiopathological effect of vascular smooth muscle hyperplasia on our experimental guinea pigs, systemic and pulmonary arterial pressures were evaluated. The experimental and control groups did not show differences in systemic or pulmonary arterial pressures (*n* = 4 in control, and *n* = 8 in asthma group; Figure 7C and D). Vascular smooth muscle hyperplasia is observed in asthma patients [44], but this hyperplasia is unrelated to pulmonary hypertension because it includes not only hyperplasia of the smooth muscle but also enhanced vascular contractility and impaired vasodilation [45].

## Conclusions

Our results suggest that the development of antigen-induced airway obstruction and hyperresponsiveness is associated with caveolin-1 expression in airway smooth muscle in a guinea pig model of asthma. It appears that the model induces the development of two different phenotypes, one contractile in airway smooth muscle and the other proliferative in pulmonary vessels. In addition, although the asthma model induced a strong caveolin-1 downregulation in vascular smooth muscle accompanied by myocyte proliferation, this phenomenon did not induce pathophysiological consequences such as changes in arterial pressure.
